# Electroacupuncture Stimulation Alleviates CFA-Induced Inflammatory Pain Via Suppressing P2X3 Expression

**DOI:** 10.3390/ijms20133248

**Published:** 2019-07-02

**Authors:** Xuaner Xiang, Sisi Wang, Fangbing Shao, Junfan Fang, Yingling Xu, Wen Wang, Haiju Sun, Xiaodong Liu, Junying Du, Jianqiao Fang

**Affiliations:** 1 Key Laboratory of Acupuncture and Neurology of Zhejiang Province, Department of Neurobiology and Acupuncture Research, The Third Clinical Medical College, Zhejiang Chinese Medical University, Hangzhou 310053, China; 2 Department of Anaesthesia and Intensive Care, The Chinese University of Hong Kong, Hong Kong SAR, China

**Keywords:** electroacupuncture, alleviates, inflammatory pain, DRG, SCDH, P2X3

## Abstract

Chronic inflammatory pain is one of the most common complaints that seriously affects patients’ quality of life. Previous studies have demonstrated that the analgesic effect of electroacupuncture (EA) stimulation on inflammatory pain is related to its frequency. In this study, we focused on whether the analgesic effects of EA are related to the period of stimulation. Purinergic receptor P2X3 (P2X3) is involved in the pathological process underlying chronic inflammatory pain and neuropathic pain. We hypothesized that 100 Hz EA stimulation alleviated Freund’s complete adjuvant (CFA) induced inflammatory pain via regulating P2X3 expression in the dorsal root ganglion (DRG) and/or spinal cord dorsal horn (SCDH). We also assumed that the analgesic effect of EA might be related to the period of stimulation. We found that both short-term (three day) and long-term (14 day) 100 Hz EA stimulation effectively increased the paw withdrawal threshold (PWT) and reversed the elevation of P2X3 in the DRG and SCDH of CFA rats. However, the analgesic effects of 100 Hz EA were not dependent on the period of stimulation. Moreover, P2X3 inhibition or activation may contribute to or attenuate the analgesic effects of 100 Hz EA on CFA-induced inflammatory pain. This result indicated that EA reduced pain hypersensitivity through P2X3 modulation.

## 1. Introduction

Inflammatory pain is a common clinical symptom that widely exists in various acute and chronic diseases [1]. However, its underlying mechanism is still unclear. At present, anti-inflammatory and analgesic drugs are commonly used in the clinic, but drug treatment is accompanied by gastrointestinal discomfort and other side effects [2].

Electroacupuncture (EA), a modified technique based on the theory of traditional manual acupuncture, has been widely used in clinical and scientific research [3,4]. Acupoints, frequency and the period of stimulation are three main elements that affect the effect of EA. Different frequencies of EA stimulation at the same acupoint may lead to different therapeutic effects, while the same frequency may also exert “pain-type” specific analgesic effects on different pathological conditions [5,6]. Our previous studies compared the analgesic effects of 2, 100 and 2/100 Hz EA on chronic pain models of inflammatory pain and neuropathic pain, e.g., Freund’s complete adjuvant (CFA) injection and spared nerve injury (SNI), respectively. We found that 100 Hz exhibited the best analgesic effect on inflammatory pain [7]. However, whether the analgesic effects of EA are related to the period of stimulation is largely unknown.

P2X3 is purinoceptor with ion channel activity in response to extracellular adenosine triphosphate (ATP) [8,9]. P2X3 is highly expressed in small- and medium-sized nociceptors of dorsal root ganglions (DRGs) and has been associated with pain response in multiple animal pain models, including the bone cancer pain model, chronic constriction nerve injury (CCI) model, spared nerve injury (SNI) model, and the CFA model [10,11,12,13]. P2X3 can be found in the peripheral terminals where it senses the ATP leaked from damaged tissues or released from inflammatory cells [14]. In addition, P2X3 is distributed in the central terminals of nociceptors, indicating that P2X3 might play presynaptic roles in the spinal cord dorsal horn [15]. Indeed, blockage of P2X3 at the spinal cord level significantly reduced postsynaptic neuronal hyperexcitability in a model of bone cancer pain [16]. Previous studies demonstrated that the expression of P2X3 was upregulated by CFA induced inflammation and CCI in the DRG and spinal cord dorsal horn (SCDH), respectively [7,17]. Notably, EA stimulation was reported to attenuate CFA (100 Hz) and CCI (15 Hz) induced pain hypersensitivity [18,19]. We speculated that EA stimulation modulated P2X3 levels in pain pathways and then exerted analgesic actions on chronic inflammatory pain.

In this study, the analgesic effects of short-term and long-term 100 Hz EA stimulation on CFA-induced chronic inflammatory pain were detected and compared. The potential involvement of P2X3 underlying EA mediated analgesia was also explored. 

## 2. Results

### 2.1. Both Short-Term and Long-Term EA Stimulation Attenuated CFA Induced Mechanical Allodynia

**Figure 1:**
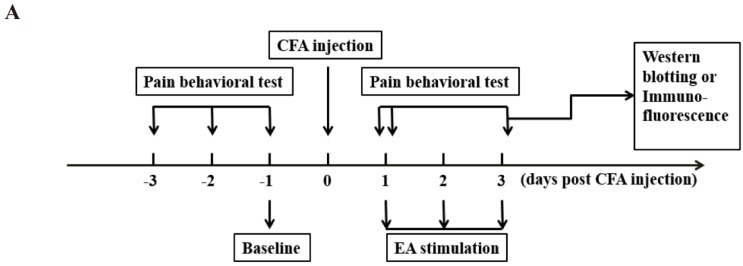

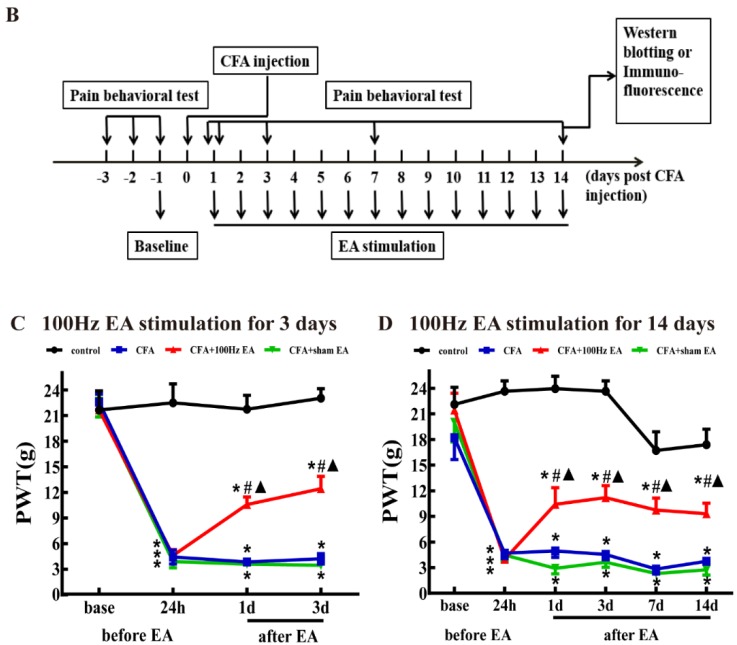
The analgesic effect of 3 days and 14 days 100 Hz electroacupuncture (EA) stimulation on the paw withdrawal threshold (PWT). (**A**) The procedure of short-term (3 days) high frequency EA stimulation experiment. (**B**) The procedure of long-term (14 days) high frequency EA stimulation experiment. (**C**) The analgesic effect of 3 days 100 Hz EA stimulation on the PWT. (**D**) The analgesic effect of 14 days 100 Hz EA stimulation on the PWT. Data are presented as the mean ± SEM, *n* = 9. * *P* < 0.05, compared with the control group; # *P* < 0.05, compared with the Freund’s complete adjuvant (CFA) group; ▲ *P* < 0.05, compared with the CFA + sham EA group.

Rats were injected with CFA into the surface of the right hindpaw to induce persistent inflammatory pain. The paw withdrawal threshold (PWT) of the rats was significantly decreased after CFA injection, indicative of mechanical allodynia establishment. The pain hypersensitivity was sustained throughout the experiment (*P* < 0.05, Figure 1). With daily EA stimulation for 3 days, 100 Hz EA significantly increased the PWT on days 1 and 3 when compared with the CFA group and sham EA group at the same time points (*P* < 0.05, Figure 1C). With daily EA stimulation for 14 days, 100 Hz EA significantly increased the PWT on days 1, 3, 7, and 14 when compared with the CFA group and sham EA group at the same time point (*P* < 0.05, Figure 1D). Compared with the no acupuncture group (i.e., the CFA only group), sham EA had no significant effect on the PWT.

### 2.2. Both Short-Term and Long-Term 100 Hz EA Stimulation Reversed P2X3 Elevation in L4-6 DRG and SCDH after CFA Injection

**Figure 2:**
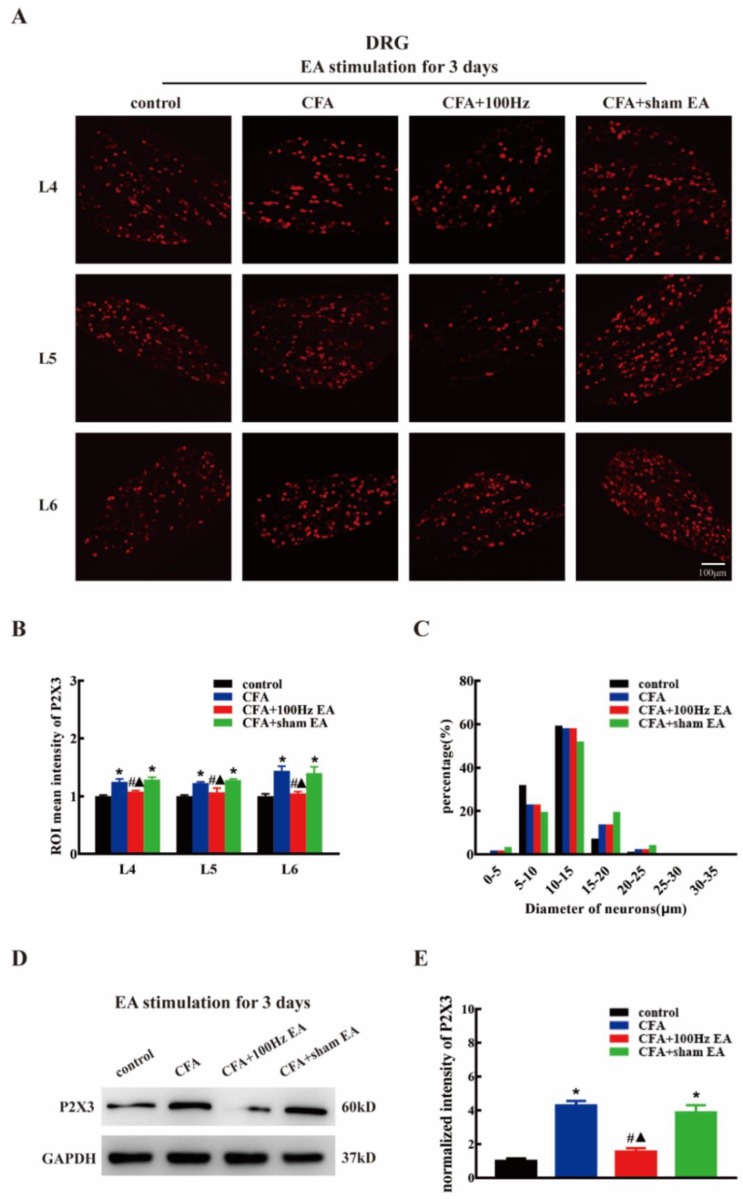
The 100 Hz EA stimulation for 3 days repressed the up-regulation of P2X3 in the L4-6 dorsal root ganglions (DRG) of inflamed rats. (**A**) Representative images of L4-6 DRG immunofluorescence staining from the control, CFA, CFA+100 Hz EA and CFA + sham EA groups. Scale bars = 100 μm. (**B**) Mean intensity analysis of P2X3-ir in L4-6 DRG in each group. Data are presented as the mean ± SEM, *n* = 3. (**C**) Size distribution of P2X3 in L4-6 DRG in different groups. (**D**) Representative western blotting images of L4-6 DRG in each group. E. Relative protein level of P2X3 in rat L4-6 DRG from different groups. Data are presented as the mean ± SEM, *n* = 6. * *P* < 0.05, compared with the control group; # *P* < 0.05, compared with the CFA group; ^▲^ *P* < 0.05, compared with the CFA + sham EA group.

**Figure 3:**
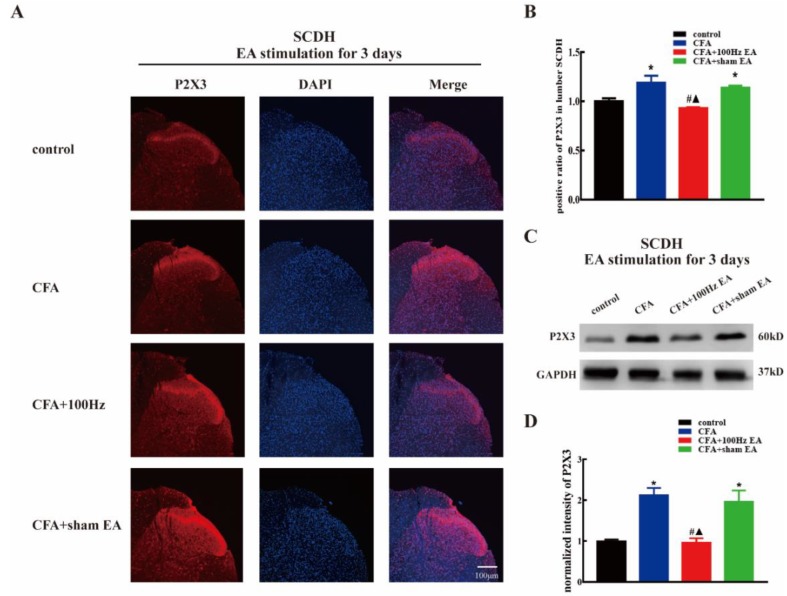
The 100Hz EA stimulation for 3 days inhibited the up-regulation of P2X3 in (spinal cord dorsal horn) SCDH of inflamed rats. (**A**). Representative images of SCDH immunofluorescence staining from the control, CFA, CFA + 100 Hz EA and CFA + sham EA groups. Scale bars = 100 μm. (**B**) Mean intensity analysis of P2X3-ir in SCDH in each group. Data are presented as the mean ± SEM, *n* = 3. (**C**) Representative western blotting images of SCDH in each group. (**D**) Relative protein level of P2X3 in SCDH from different groups. Data are presented as the mean ± SEM, *n* = 6. * *P* <0.05, compared with the control group; # *P* <0.05, compared with the CFA group; ^▲^ *P* <0.05, compared with the CFA + sham EA group.

**Figure 4:**
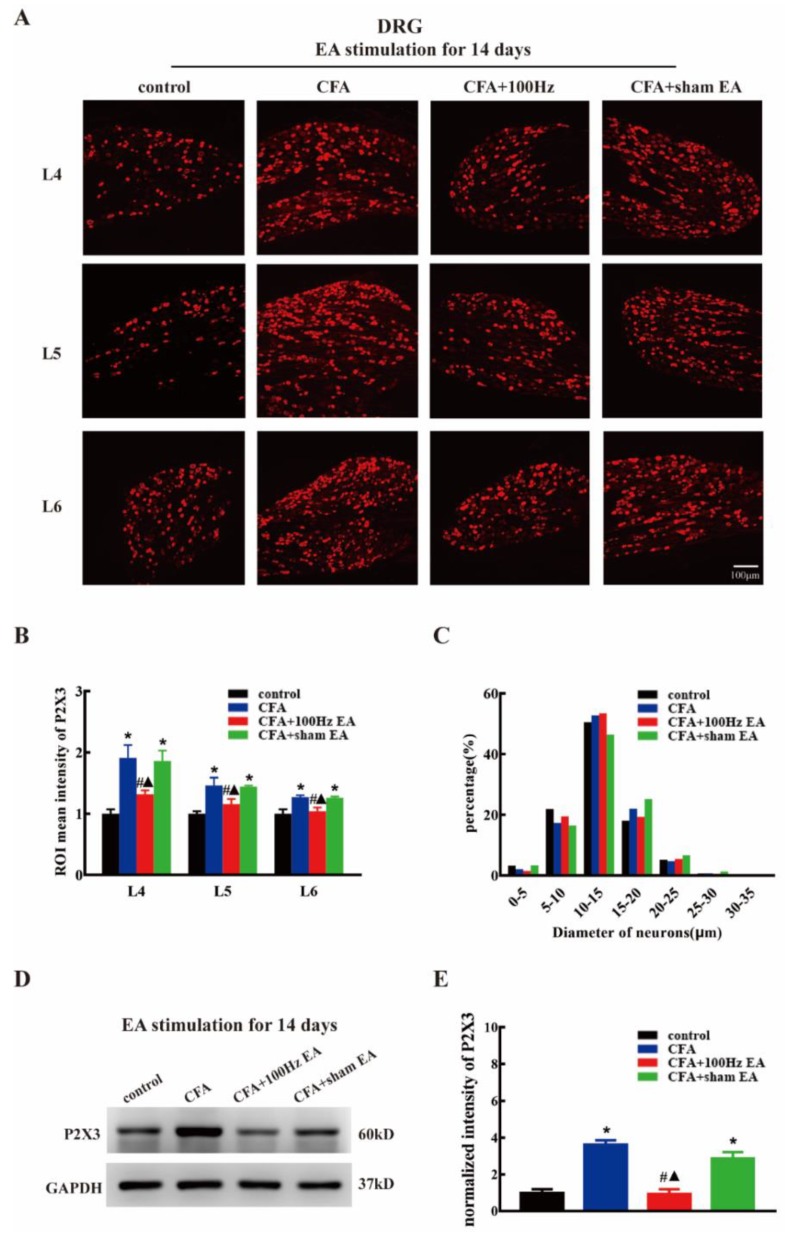
The 100Hz EA stimulation for 14 days repressed the up-regulation of P2X3 in the L4-6 DRG of inflamed rats. (**A**) Representative images of L4-6 DRG immunofluorescence staining from the control, CFA, CFA + 100 Hz EA and CFA + sham EA groups. Scale bars = 100 μm. (**B**) Mean intensity analysis of P2X3-ir in L4-6 DRG in each group. Data are presented as the mean ± SEM, *n* = 3. (**C**) Size distribution of P2X3 in L4-6 DRG in different groups. (**D**) Representative western blotting images of L4-6 DRG in each group. (**E**) Relative protein level of P2X3 in rat L4-6 DRG from different groups. Data are presented as the mean ± SEM, *n* = 6. * *P* < 0.05, compared with the control group; # *P* <0.05, compared with the CFA group; ^▲^ *P* <0.05, compared with the CFA + sham EA group.

**Figure 5:**
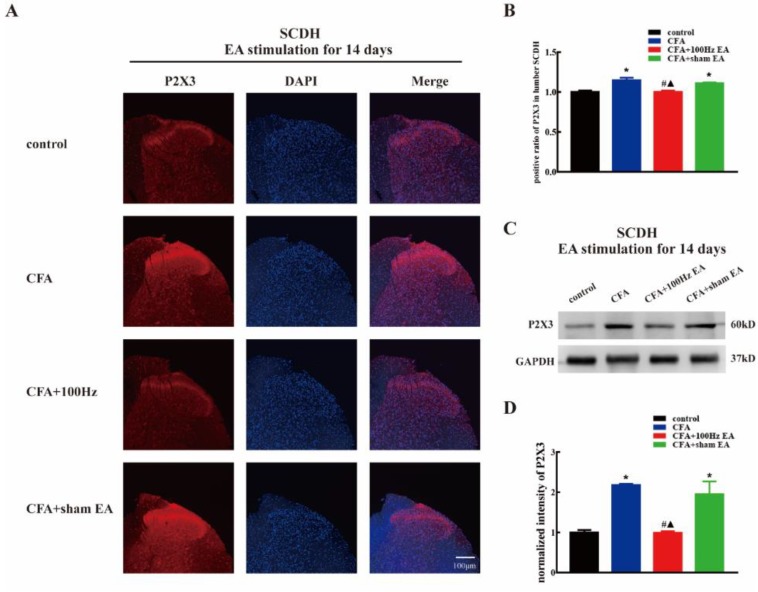
The 100 Hz EA stimulation for 14 days inhibited the up-regulation of P2X3 in the SCDH of inflamed rats. (**A**) Representative images of SCDH immunofluorescence staining from the control, CFA, CFA + 100 Hz EA and CFA + sham EA groups. Scale bars = 100 μm. (**B**) Mean intensity analysis of P2X3-ir in the SCDH in each group. Data are presented as the mean ± SEM, *n* = 3. (**C**) Representative western blotting images of SCDH in each group. (**D**) Relative protein level of P2X3 in the SCDH from different groups. Data are presented as the mean ± SEM, *n* = 6. * *P* <0.05, compared with the control group; # *P* < 0.05, compared with the CFA group; ^▲^ *P* < 0.05, compared with the CFA + sham EA group.

To investigate the effects of the short-term and long-term 100 Hz EA stimulation on the expression of P2X3 in the DRG and SCDH following CFA injection, we used immunofluorescence and western blotting to measure the P2X3 protein levels in the DRG and SCDH in the control group, CFA group, 100 Hz EA group and sham EA group, 3 or 14 days after 100 Hz EA stimulation. As expected, CFA injection significantly increased the mean intensity of P2X3-ir in L4-6 DRG (Figure 2A,B, *P* < 0.05; Figure 4A,B, *P* < 0.05) and SCDH (Figure 3A,B, *P* < 0.05; Figure 5A,B, *P* < 0.05). Short-term and long-term 100 Hz EA stimulation significantly reduced the mean intensity of P2X3-ir in L4-6 DRG (Figure 2A,B, *P* < 0.05; Figure 4A,B, *P* < 0.05) and SCDH (Figure 3A,B, *P* < 0.05; Figure 5A,B, *P* < 0.05) when compared with the control group. In contrast, sham EA had no observable effects. As shown in Figure 2C and Figure 4C, P2X3 was mainly expressed in the small- and medium- diameter DRG neurons (diameter less than 35 μm). Neither short-term nor long-term 100 Hz EA changed the distribution of P2X3.

We also used western blotting to measure the P2X3 protein expression. CFA injection increased P2X3 protein expression in L4-6 DRG (Figure 2D,E, *P* < 0.05; Figure 4D,E, *P* < 0.05) and SCDH (Figure 3C,D, *P* < 0.05; Figure 5C,D, *P* < 0.05). Consistent with the immunofluorescence staining results, short-term and long-term 100 Hz EA stimulation significantly reversed paw inflammation induced P2X3 up-regulation in L4-6 DRG (Figure 2D,E, *P* < 0.05; Figure 4D,E, *P* < 0.05) and SCDH (Figure 3C,D, *P* < 0.05; Figure 5C,D, *P* < 0.05). Sham EA had no effect on P2X3 expression either in L4-6 DRG or SCDH.

### 2.3. Short-Term and Long-Term EA Stimulation Exerted Comparable Effects on Pain Hypersensitivity and P2X3 Expression

**Figure 6:**
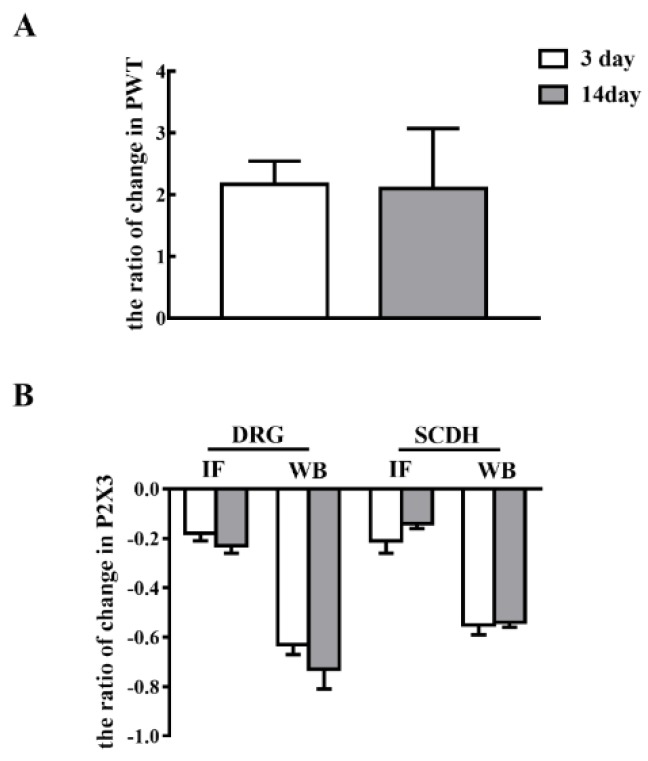
Short-term and long-term EA stimulation treatments exerted comparable effects on pain hypersensitivity and P2X3 expression. (**A**) The ratio of changes in PWT after 100 Hz EA stimulation for 3 and 14 days. (**B**) The ratio of changes in P2X3 after 100 Hz EA stimulation for 3 and 14 days. Data are presented as the mean ± SEM.

To investigate the effects of short-term and long-term EA stimulation, we compared the ratio of change in PWT and P2X3 expression in L4-6 DRG and SCDH. As shown in Figure 6A, there was no significant difference in the ratio of changes in PWT between EA stimulation for 3 and 14 days (*P* > 0.05). Additionally, there was no significant difference in the ratio of changes in P2X3 expression either in L4-6 DRG or SCDH between EA stimulation for 3 and 14 days (Figure 6B, *P* > 0.05).

### 2.4. P2X3 Levels in L4-6 DRG and SCDH Are Involved in Chronic Inflammatory Pain

**Figure 7:**
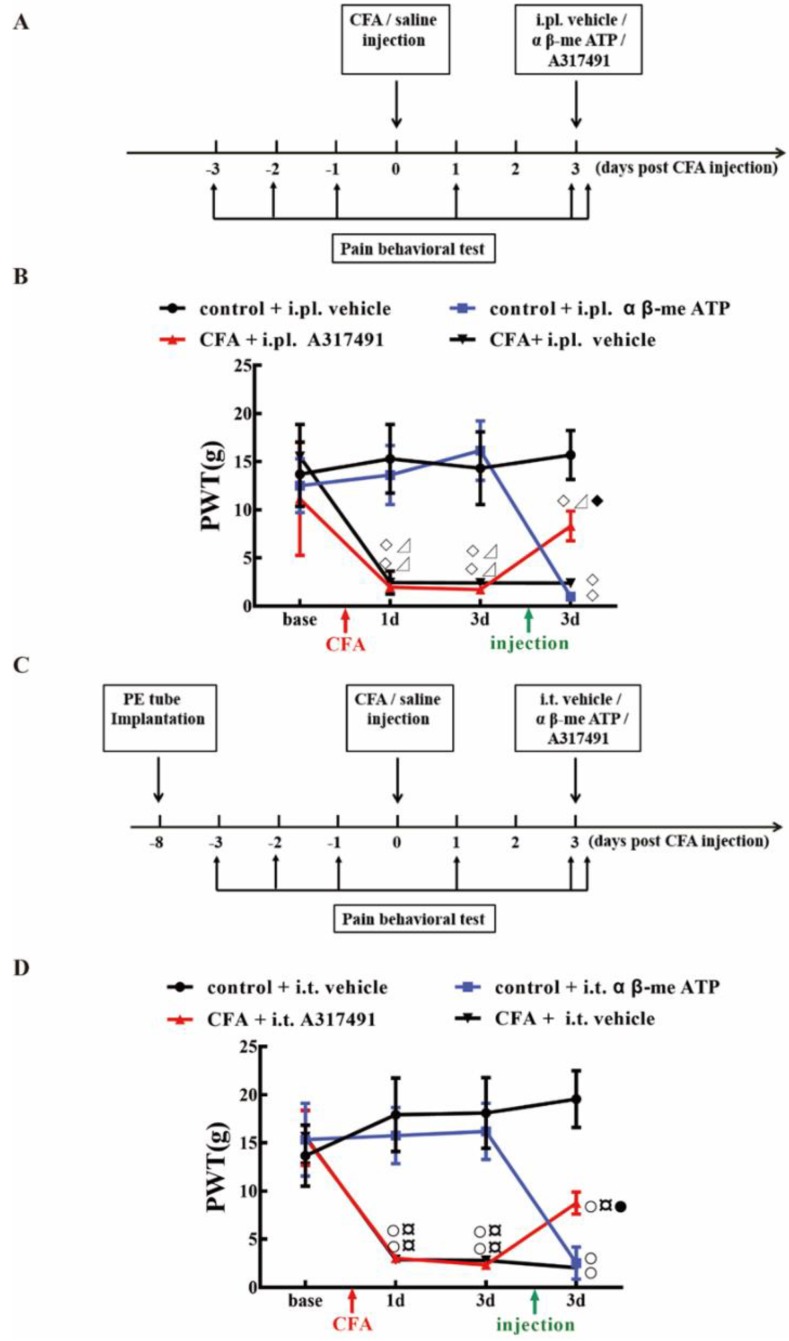
The effect of intraplantar injection or intrathecal injection of α β-me ATP (P2X3 agonist) and A317491 (P2X3 antagonist) on the PWT. (**A**) Schematic flow diagram of the intraplantar injection of α β-me ATP (P2X3 agonist) and A317491 (P2X3 antagonist). (**B**) The effect of intraplantar injection of α β-me ATP (P2X3 agonist) and A317491 (P2X3 antagonist) on the PWT. Data are presented as the mean ± SEM, *n* = 5. ◇ *P* < 0.05, compared with the control + intraplantar injection (i.pl.) vehicle; ⊿ *P* < 0.05, compared with the control + i.pl. α β-me ATP; ^◆^ *P* < 0.05, compared with the CFA + i.pl. vehicle group. (**C**) Schematic flow diagram of the intrathecal injection of α β-me ATP (P2X3 agonist) and A317491 (P2X3 antagonist). (**D**)The effect of intrathecal injection of α β-me ATP (P2X3 agonist) and A317491 (P2X3 antagonist) on the PWT. Data are presented as the mean ± SEM, *n* = 5. ^○^ *P* < 0.05, compared with the control + intrathecal injection (i.t.) vehicle; ^¤^ *P* <0.05, compared with the control + i.t. α β-me ATP group; ^●^ *P* < 0.05, compared with the CFA + i.t. vehicle.

Previous studies demonstrated that the P2X3 agonist αβ-me ATP might induce pain hypersensitivity in rats. In this study, intraplantar injection (i.pl.) or intrathecal injection (i.t.) of αβ-me ATP can induce mechanical hyperalgesia in normal rats. Compared with the control + vehicle, the PWTs of control + i.pl. α β-me ATP, and control + i.t. α β-me ATP both decreased rapidly after αβ-me ATP administration (Figure 7B,D, *P* < 0.05). In contrast, treatment with A317491 (P2X3 antagonist) via i.pl. or i.t., significantly alleviated CFA induced mechanical hypersensitivity (Figure 7B,D). These outcomes suggested that P2X3 activation is sufficient to cause pain hypersensitivity, and that P2X3 might play essential roles in the modulation of pain signal transmission from the DRG to the spinal cord. 

### 2.5. P2X3 Inhibition Contributed to the Analgesic Effects of 100 Hz EA on CFA-Induced Inflammatory Pain

**Figure 8:**
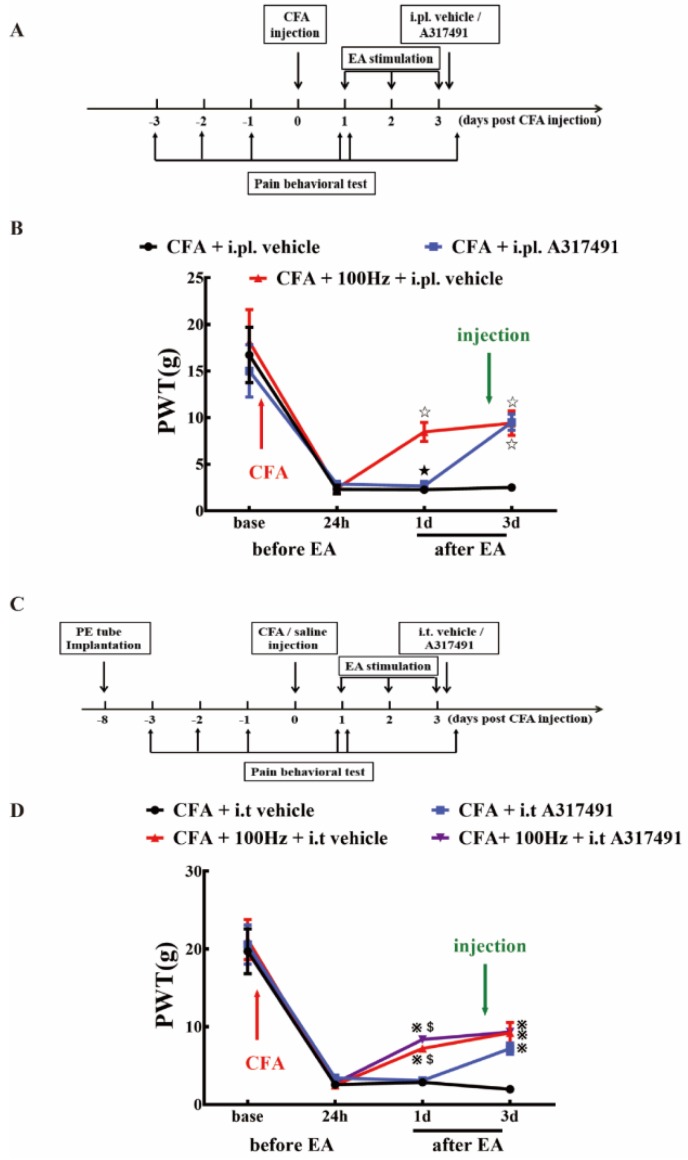
P2X3 inhibition contributed to the analgesic effects of 100 Hz EA on CFA-induced inflammatory pain. (**A**) Schematic flow diagram of the intraplantar injection of P2X3 antagonist A317491. (**B**) The effect of intraplantar injection of A317491 on the PWT. Data are presented as the mean ± SEM, *n* = 6. ^☆^ *P* < 0.05, compared with the CFA + i.pl. vehicle group; ^★^ *P* < 0.05, compared with the CFA + 100 Hz + i.pl. vehicle group. (**C**) Schematic flow diagram of the intrathecal injection of A317491. (**D**) The effect of intrathecal injection of A317491on the PWT. Data are presented as the mean ± SEM, *n* = 6. ^※^ *P* < 0.05, compared with the CFA + i.t vehicle group; **^$^***P* < 0.05, compared with the CFA + 100 Hz+ i.t vehicle group.

To investigate whether P2X3 is involved in the analgesic effect of 100 Hz EA on chronic inflammatory pain, A317491 was co-administered via i.pl. and i.t. to observe the changes in PWT in rats of each group. Consistent with the findings in Figure 7, i.pl. and i.t. of A317491 exerted significant analgesic effects on CFA induced inflammatory pain (Figure 8B,D). Similarly, EA stimulation also efficiently alleviated CFA induced mechanical allodynia, as indicated by the results of CFA + i.pl. vehicle versus CFA + 100 Hz + i.pl. vehicle (Figure 8B) and CFA + i.t. vehicle versus CFA + 100 Hz + i.t. vehicle (Figure 8D). Notably, co-treatment with EA and i.t. A317491 did not further increase the analgesia compared with EA alone (Figure 8D).

**Figure 9:**
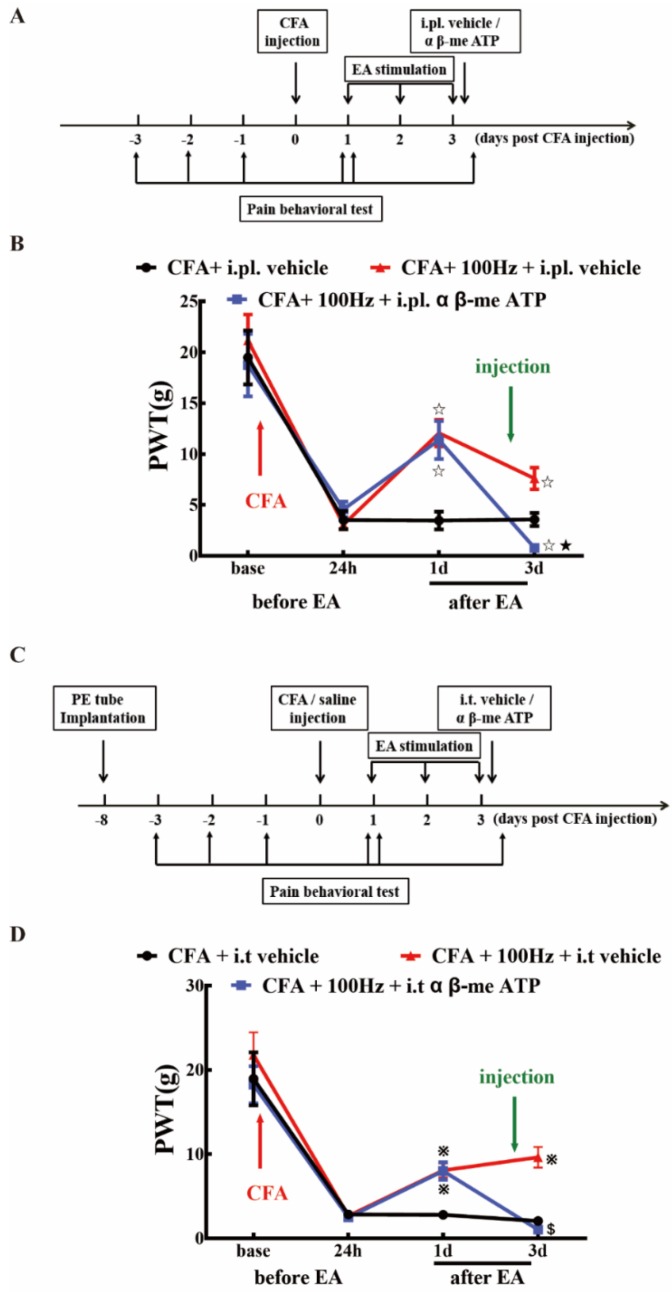
α β-me ATP (P2X3 agonist) attenuated the analgesic effect of 100 Hz EA on PWT in CFA rats. (**A**) Schematic flow diagram of the intraplantar injection of α β-me ATP. (**B**) The analgesic effect of 100Hz EA can be attenuated by intraplantar injection of α β-me ATP. Data are presented as the mean ± SEM, *n* = 6. ^☆^ *P* < 0.05, compared with the CFA + i.pl. vehicle group; ^★^ *P* < 0.05, compared with the CFA + 100Hz + i.pl. vehicle group. (**C**) Schematic flow diagram of the intrathecal injection of α β-me ATP. (**D**) Intrathecal injection of α β-me ATP may reduce the analgesic effect of 100 Hz EA. Data are presented as the mean ± SEM, *n* = 6. ^※^ *P* < 0.05, compared with the CFA + i.t vehicle group; **^$^***P* < 0.05, compared with the CFA + 100Hz + i.t vehicle group.

To further confirm that 100 Hz EA has an analgesic effect on chronic inflammatory pain by regulating P2X3, α β-me ATP was administered via i.pl. and i.t. to observe the changes in PWT in rats of each group. As shown in Figure 9, i.pl. and i.t. of α β-me ATP can reverse the analgesic effect of electroacupuncture, as indicated by the results of the CFA + 100 Hz + i.pl. vehicle group versus the CFA + 100 Hz + i.pl. α β-me ATP group (Figure 9B) and the CFA + 100 Hz + i.t. vehicle group versus the CFA + 100 Hz + i.t. α β-me ATP group (Figure 9D). This result provided evidence that EA reduced pain hypersensitivity through P2X3 modulation.

## 3. Discussion

The frequency of EA stimulation appears to be a determinant of the analgesic effect of EA [20]. The optimal frequency of EA treatment is not constant in different types of pathological pain [21]. While both 2 and 100 Hz EA treatment relieved type 2 diabetic neuropathic pain, 2 Hz exerted stronger analgesic effects 100 Hz [22]. However, we have demonstrated that the analgesic effect of EA was greater at 100 Hz than at 2 Hz in the scenario of inflammatory pain [7]. Similarly, 100 Hz, but not 2 Hz EA stimulation, could relieve post-incision pain [23]. In addition to the frequency, the number of stimulations may also significantly affect the analgesic effects of EA, indicative of the presence of a cumulative effect [24,25]. However, in the current study, we observed that daily EA stimulations for 3 days and 14 days provided comparable analgesia for persistent inflammatory pain. We concluded that a “ceiling” effect can occur during the application of EA stimulation. This finding may help determine the regimen of EA application for the treatment of chronic inflammatory pain. 

Clinical studies and scientific studies have proved that EA has eminent analgesic effects, but the mechanism of EA analgesia is still an open question [26,27,28]. Cheng RS et al. reported that 4 Hz EA attenuated pain through the modulation of endorphins, whereas the analgesic effect of 200 Hz EA may be mediated through serotonin [29]. Wang Y et al. demonstrated that 100 Hz EA relieved inflammatory pain by increasing CXCL10, which chemoattracted opioid-containing macrophages and mediated the anti-nociceptive effect in the model of inflammatory pain [30]. Kim H W et al. proposed that 1 Hz electroacupuncture suppressed carrageenan-induced paw inflammation via sympathetic post-ganglionic neurons, while inflammation was restrained by 120 Hz EA in connection with the sympathoadrenal medullary axis [31]. Recently, increasing evidence has shown that the analgesic effect of EA is closely related to its regulation of ion channels in sensory neurons [32,33]. Particularly, increased attention has been paid to P2X3, which has been regarded as a potential target of inflammatory pain and neuropathic pain [34,35].

Inhibition of the P2X3 receptor through a selective antagonist, e.g., A317491 has been assessed as a potential approach for inflammatory pain management [36,37]. Using the model of CFA induced chronic inflammation, Qian Jiang et al. reported that the expression of P2X3 was markedly increased in DRG tissues [13]. Similar results were obtained in the current study. On the 3rd and 14th days after CFA injection, the expression of P2X3 was significantly increased in L4-6 DRG as indicated by immunofluorescence staining and western blotting. We confirmed that P2X3 was distributed in small- and medium-sized neurons, especially in the diameter range of 5–10 μm. Several studies shown that EA can be applied to treat different types of pain (such as neuropathic pain, inflammatory pain, and bone cancer pain) by down-regulating DRG P2X3 [34,38,39]. Consistently, we demonstrated that both short-term and long-term EA stimulation can effectively down-regulate the up-regulation of P2X3 in DRG induced by CFA. Few studies have focused on the expression of the P2X3 in the SCDH of CFA model. In this study, we found that the mean intensity of immunoreactivity and protein level of the P2X3 in the SCDH were significantly increased at three days and 14 days after CFA injection. In relation to DRG, elevation of P2X3 was also reversed by short-term and long-term EA in SCDH. Notably, that P2X3 is distributed in the presynaptic part of the spinal cord, i.e., the central terminal of nociceptors. Therefore, EA mediated inhibition of spinal P2X3 is relies on its effects on the DRG. The current findings suggested that EA might directly modulate pain signal transmission from first-order neurons (DRG) to second-order neurons (SCDH). Indeed, post-treatment with P2X3 inhibitor via intrathecal injection failed to further enhance the anti-nociceptive effects by EA, indicating that spinal P2X3 played essential roles underlying EA mediated analgesia. While overexpression of DRG and SCDH P2X3 in CFA rats nearly returned to normal levels after EA stimulation, but the hyperalgesia of CFA rats still existed despite relief. The reason for this phenomenon may be due to proinflammatory cytokines, NLRP, CB2, and other substances that are involved in CFA-induced chronic inflammatory pain. P2X3 is not the only determinant, even though it plays an important role in the occurrence and maintenance of chronic inflammatory pain [40,41,42]. 

α β-me ATP and A317491 were co-administered via i.pl. and i.t. to further prove that P2X3 is involved in the pathological process of chronic inflammatory pain. We found that α β-me ATP injection in normal rats can induce hyperalgesia, while A317491 injection in CFA rats can effectively reverse CFA-induced hyperalgesia, indicating that P2X3 is closely related to inflammatory pain induced by CFA. Then A317491 was administered, and the analgesic effect was consistent with 100 Hz EA, and effectively relieved CFA-induced mechanical hyperalgesia. However, α β-me ATP administered via i.pl. and i.t. effectively reduced the analgesic effect of 100 Hz EA. This finding reveals that P2X3 regulation may be a potential mechanism for the analgesic effect of EA.

We compared the ratio of change in the PWT and the P2X3 expression in DRG and SCDH between short-term and long-term EA stimulation in CFA rats. We discovered that the ratio of change in DRG P2X3 in long-term EA stimulation was slightly higher than that in short-term EA stimulation, but there was no significant difference. However, the ratio of change in SCDH P2X3 in short-term EA stimulation was slightly higher than that in long-term EA stimulation, and the difference was not significant. Therefore, we speculated that the analgesic effect of EA on chronic inflammatory pain may not be related to the term of stimulation.

## 4. Materials and Methods

### 4.1. Animals

Male Sprague-Dawley (SD) rats (180–220 g) were purchased from the Experimental Animal Center of Zhejiang Chinese Medical University. All rats in this experiment were housed in a controlled environment (five rats per cage, temperature: 25 ± 2 °C, humidity: 55% ± 5%, and light: 12 h light/dark cycle) and were fed a standard rodent food and allowed distilled water ad libitum. All experimental procedures were approved by the Animal Care and Welfare Committee of Zhejiang Chinese Medical University, Zhejiang, China (ZSLL, 2015-022).

### 4.2. Experimental Design

#### This study was divided into three parts.

In part one, the effects of short-term or long-term 100 Hz EA stimulation on CFA induced pain were monitored. For short-term EA stimulation experiments, rats were randomly assigned into four groups (*n* = 9/group): (1) control group, (2) CFA group, (3) CFA + EA group, and (4) CFA + sham EA group. Rats were treated by acupuncture with (EA) or without (sham EA) 100 Hz electric current stimulation for 3 days after CFA injection. Pain behavioral tests were conducted according to the schedule (Figure 1A), i.e., on days -3, -2, -1, and zero before EA stimulation, and on days 1 and 3 after EA stimulation, respectively. After 3 days post EA stimulation, rats were sacrificed for tissue collection. The L4–6 DRG and lumbar spinal cord were removed for immunofluorescence staining or western blotting. For long-term EA stimulation experiments, rats were assigned to four groups as above, and administered with EA or sham EA for 14 days after CFA injection. Pain behavioral tests were conducted according to the schedule (Figure 1B), i.e., on days -3, -2, -1 and zero before EA stimulation, and on days 1, 3, 7 and 14 after EA stimulation. After fourteen days post EA stimulation, tissues were collected and applied as above. 

In part two, the involvement of P2X3 in EA-mediated pain modulation was explored through the administration of a P2X3 antagonist (A317491) or agonist (α β-me ATP) via intraplantar injection (i.pl.) or intrathecal injection (i.t.). Rats were randomly assigned to eight groups (*n* = 5/group), i.e., (1) control + i.pl. vehicle group, (2) control + i.pl. α β-me ATP group, (3) CFA + i.pl. A317491 group, (4) CFA + i.pl. vehicle group, (5) control + i.t. vehicle group, (6) control + i.t. α β-me ATP group, (7) CFA + i.t. A317491 group, and (8) CFA + i.t. vehicle group. For i.pl., antagonists or agonists were administered ipsilaterally on day three after CFA injection, and paw withdrawal thresholds (PWT) were recorded according to the schedule (Figure 7A). For i.t., antagonists or agonists were delivered via implanted PE tubes. PWT was recorded according to the schedule (Figure 7C).

In part three, to explore the role of P2X3 in DRG and SCDH on the analgesic effect of 100 Hz EA stimulation on CFA rats, we investigated whether peripheral subcutaneous injection or central intrathecal injection of P2X3 inhibitors could simulate the analgesic effect of 100 Hz EA (Figure 8). A group of 42 adult male rats (*n* = 6/group) were divided into a (1) CFA + i.pl. vehicle group, (2) CFA + i.pl. A317491 group, (3) CFA + 100 Hz + i.pl. vehicle group, (4) CFA + i.t. vehicle group, (5) CFA + i.t. A317491 group, (6) CFA + 100 Hz + i.t. vehicle group, and a (7) CFA + 100 Hz + i.t. A317491 group. Then, we observed whether peripheral subcutaneous injection or central intrathecal injection of a P2X3 agonist (α β-me ATP) could reverse the analgesic effect of 100 Hz EA (Figure 9). We randomly assigned 36 adult male rats to six groups, including a (1) CFA + i.pl. vehicle group, (2) CFA + 100 Hz + i.pl. α β-me ATP group, (3) CFA + 100 Hz + i.pl. vehicle group, (4) CFA + i.t. vehicle group, (5) CFA + 100 Hz + i.t. α β-me ATP group, and a (6) CFA + 100 Hz + i.t. vehicle group. 

### 4.3. Persistent Inflammatory Pain Model

Persistent inflammatory pain like responses were induced via i.pl. of 0.1mL CFA (Sigma-Aldrich, St. Louis, MO, USA) into the plantar surface of the right hind paws of SD rats. For the sham control, saline was applied via i.pl.

### 4.4. Paw Withdraw Threshold (PWT)

PWT was determined by the von Frey behavioral test, which was performed according to the up-down method described by Chaplan et al. [43]. Rats were placed in the individual testing cages for 30 min per day for three continuous days to adapt to the test environment. Before each test, rats were placed into the cage for at least 15 min to acclimate to the environment. The von Frey hairs (Stoelting Co, Thermo, Gilroy, CA, USA) were applied in a consecutive ascending order (0.4, 0.6, 1, 2, 4, 6, 8, 15, and 26 g) to the central surface of the hind paw and sustained for 5 s. The first hair applied corresponded to a force of 4 g. Brisk withdrawal or paw flinching was considered a positive response and marked as “X”. A weaker stimulus was then applied. In the case of no responses, “O” was recorded, followed by a stronger stimulus. The interval between each stimulus was not less than 2 min. After the combination of “OX” or “XO” appeared, a series of four stimuli were applied and recorded as above. The 50% PWTs of the rats were calculated by the formula 50% PWTs (g) = 10 ^ (xf + k *δ- 4). “xf” is the logarithmic value of the last von Frey hair in the sequence, “k” is the corresponding value of the resulting sequence in the k-value table, and “δ” is the mean difference of each filament strength after logging (0.231 in the current cases). If a positive stimulus appeared five consecutive times, PWT was marked as 26 g. If five “X”s were recorded, PWT was marked as 0.4 g. If the value of 50% PWTs was greater than 26 g, 26 g was used as the maximum. And if the value of 50% PWTs was less than 0.4 g, 0.4 g was used as the minimum. The measuring time was fixed at 9:00–16:00, and the ambient temperature was 23 ± 2 °C.

### 4.5. EA Treatment

All rats in CFA + 100 Hz EA group were treated with the EA stimulus. Zusanli (ST36) and Kunlun (BL60) acupoints were taken from the bilateral legs of the rats. Acupuncture needles, 0.25 mm * 13 mm were used in this study. The needles were inserted into the acupoints at a depth of 5 mm and then stimulated by HANS Acupuncture Point Nerve Stimulator (HANS-200A Huawei Co., Ltd., Beijing, China). The parameters of the stimulator were as follows: 100 Hz, 0.5–1.5 mA (initial strength 0.5 mA, increased by 0.5 mA every 10 min) for a total of 30 min. The stimulus was conducted once daily in a period of 3 or 14 days. The rats in the CFA group were only given the same fixed time as the EA group. No treatment was performed in the control group. The CFA + sham EA group animals received needle insertion subcutaneously into ST36 and BL60 (1 mm in depth). The needles were connected to the electrodes without electrical stimulation. After finishing the EA or sham EA stimulation, the PWT was measured immediately.

### 4.6. Drug Treatment

α β-me ATP (P2X3 agonist) and A317491 (P2X3 antagonist) were purchased from Sigma-Aldrich (Sigma-Aldrich, Saint Louis, MO, USA), and dissolved in sterile 0.9 % saline solution to prepare stock solution (stored at –20 °C). They were diluted to the requested concentrations before each experiment. For i.pl, α β-me ATP (600 nmol,10 μL) and A317491 (300 nmol,10 μL) were injected subcutaneously into the dorsal surface of the right hindpaw of rats. For i.t., α β-me ATP (300 nmol, 25 μL) and A317491 (100 nmol, 25 μL) were administered once on days 3 after CFA injection.

### 4.7. Immunofluorescence

Animals were sacrificed after the behavioral testing on days 3 and 14. Rats were deeply anesthetized using pentobarbital (80 mg/kg, i.p.) and transcardially perfused with 150 mL normal saline (4°C) and 400 mL 4% paraformaldehyde in 0.1 M phosphate-buffered saline (PBS) for prefixation. The L4–6 segments of the dorsal root ganglion (DRG) and lumbar spinal cord were removed and postfixed in 4% paraformaldehyde for 3 hours at 4 °C before transfer to 15% and 30% sucrose for dehydration. Tissues were embedded in Tissue-Tek O.C.T compound (SAKURA, Torrance, CA, USA). DRGs were cut at thickness of 14 μm, and frozen sections of the lumbar spinal cord were cut at thickness of 20 μm using a CryoStar (NX50 HOP, Thermo, Walldorf, Germany).

Sections were rinsed with TBST (0.1 % Tween-20) and blocked with 5% normal donkey serum for one hour at 37 °C. Sections were then incubated with rabbit anti-P2X3 (1:800 in 5 % normal donkey serum, Alomone, Jerusalem, Israel) overnight at 4 °C. The slides were then incubated in Alexa Fluor 647-conjugated AffiniPure donkey anti-rabbit IgG (H + L) (Jackson, West Grove, PA, USA) for one hour at 37 °C. Images were taken using the A1R confocal microscope (Nikon, Tokyo, Japan). We used NIS-Elements AR to calculate the mean intensity of P2X3-immunoreactivity (-ir) in the region-of-interest (ROI) in DRG and SCDH. The relative level of P2X3 in each group was normalized to the expression in the control group.

### 4.8. Western Blotting Analysis

Animals were sacrificed after the behavioral testing on days 3 and 14. Rats were deeply anesthetized using pentobarbital (80 mg/kg, i.p.) and transcardially perfused with 150 mL normal saline (4 °C).The L4–6 segments of the DRG and lumbar spinal cord were removed and stored at −80 °C. Tissues were homogenized in strong RIPA buffer (50 mM Tris (pH 7.5), 150 mM NaCl, 1% Triton X-100, 1% sodium deoxycholate, sodium orthovanadate, 0.1% Sodium dodecyl sulfate, Ethylene Diamine Tetraacetic Acid, sodium fluoride, leupeptin, and 1 nM PMSF). The homogenate was allowed to rest on ice for 30 min and centrifuged at 15,000 rpm for 15 min at 4 °C. The supernatant was then collected for further operations. The protein concentration of tissue lysates was determined with a BCA protein assay kit. Lysates were denatured and loaded (15 μg total protein per lane). Protein samples were separated on 5–10% Sodium dodecyl sulfate-polyacrylamide gelelectrophoresis gels and electrophoretically transferred to polyvinylidene difluoride (PVDF) membranes (Merck KGaA, Darmstadt, Germany). The membranes were blocked with 5% low-fat milk in TBST for one hour at room temperature. We used rabbit anti-P2X3 (1:1000 in 5% low-fat milk, Alomone, Jerusalem, Israel) as the primary antibody and horseradish peroxidase (HRP)-conjugated goat anti-rabbit IgG as the secondary antibody (1:10,000, CST, Danvers, MA, USA). Rabbit anti-GAPDH (HRP Conjugate) (1:1000, CST, Danvers, MA, USA) was used as the internal control. The membranes were developed with an ECL kit (Pierce, Rockford, lL , USA), and the signals were captured with an Image Quant LAS 4000 (GE, Pittsburgh, PA, USA). The density of each band was measured using Image Quant TL 7.0 analysis software (GE, Pittsburgh, PA, USA). The relative level of P2X3 in each group was normalized to the expression of the control group.

### 4.9. Statistical analysis

All data are expressed as the mean ± standard error of the mean (SEM). The PWTs among groups were compared by multi-factor analysis of variance (ANOVA), followed by Bonferroni’s post hoc test to compare the significant difference between groups or between time points. All other data were analyzed by one-way ANOVA, followed by Bonferroni’s post hoc tests. *P* < 0.05 was considered statistically significant.

## 5. Conclusions

In summary, we concluded that both short-term and long-term 100 Hz EA stimulation provided significant pain relief for chronic inflammatory pain, and this analgesic effect was related to the suppression of P2X3 in the DRG and SCDH.
